# Cutaneous melanocytic tumor with *CRTC1::TRIM11* fusion: a case report

**DOI:** 10.1186/s13000-023-01437-2

**Published:** 2024-01-06

**Authors:** Rong Duan, Xiaojuan He, Xiaojing Ma, Fengbo Huang, Xiangrong Hu

**Affiliations:** Department of Pathology, Second Affiliated Hospital of Zhejiang University School of Medicine, Hangzhou, China

## Abstract

**Background:**

Cutaneous Melanocytic Tumor with *CRTC1::TRIM11* Fusion (CMTCT) represents a novel and rare entity in the realm of dermatological oncology, characterized by distinct melanocytic differentiation. This particular tumor type has yet to be officially recognized by the World Health Organization (WHO). CMTCT is generally perceived as a tumor with a relatively indolent nature; however, it is not devoid of metastatic potential. Therefore, ensuring complete surgical excision of the tumor, coupled with rigorous long-term follow-up, is paramount for patient management. In this context, we report the case of an 18-year-old female patient who presented with a dull red nodule on her left leg. Initial surgical intervention led to a pathological diagnosis of CMTCT, but it was determined that the tumor had not been fully excised. Consequently, a second surgical procedure was undertaken to achieve complete removal of the tumor. During a follow-up period of six months post-surgery, the patient showed no signs of local recurrence or metastasis, indicating a successful outcome.

**Case presentation:**

An 18-year-old female patient noticed a dull red nodule on her left leg three years ago, which exhibited slow growth over time. She underwent a subcutaneous tumor resection. Histological examination under high-power magnification revealed that the neoplasm consisted of epithelioid cells arranged in nests, fascicles, bundles, or sheets. The tumor cells had round or ovoid nuclei with prominent nucleoli and visible mitotic figures. Notably, areas resembling nevus cell clusters were observed. Immunohistochemical analysis confirmed melanocytic differentiation. Next-generation sequencing (NGS) identified a *CRTC1::TRIM11* fusion, and fluorescence in situ hybridization (FISH) for CRTC1 confirmed rearrangement. Consequently, a diagnosis of cutaneous melanocytic tumor with *CRTC1::TRIM11* fusion was established.

**Conclusions:**

CMTCT is a rare tumor characterized by melanocytic differentiation. In this case, the tumor predominantly comprised epithelioid cells with localized nevus cell clusters. The expression of melanocyte markers could easily lead to a misdiagnosis as cutaneous melanoma. However, several distinguishing features were noted: the tumor was not connected to the epidermis, exhibited low cellular heterogeneity and proliferation index, and showed minimal cellular atypia. Additionally, tests for EWSR1 rearrangement (FISH) and BRAF V600E mutation (PCR-ARMS) were negative.This case underscores the importance of a comprehensive diagnostic approach when clinical, microscopic, immunohistochemical, and molecular findings do not align. The presence of nevus cell clusters morphology in the tumor cells enhances our understanding of this disease’s histological spectrum and aids in avoiding misdiagnosis or missed diagnosis.

## Introduction

Tumors with melanocytic differentiation are important and often difficult to diagnose in surgical pathology. While the vast majority of melanocytic neoplasms are classifiable through morphological assessment, immunohistochemical studies, and molecular diagnostics, a subset remains that defies conventional classification. In 2018, Cellier et al. first reported five cases of unpigmented nodular tumors combining a melanocytic phenotype and low-grade tumor behavior with unique molecular features. They termed them as “cutaneous melanocytoma with *CRTC1::TRIM11* fusion” (CMCT) [1]. Subsequently, Bontoux et al. reported a case of a *CRTC1::TRIM11* fused neoplasm exhibiting recurrence and metastasis after 13 years, suggested that this entity represents a more aggressive cutaneous variant of CCS with a novel *CRTC1::TRIM11* fusion other than the *EWSR1::ATF1* [2]. Further expanding on this, Ko et al. presented four additional cases, proposing a nomenclature update to ‘cutaneous melanocytic tumor with *CRTC1::TRIM11* fusion’ (CMTCT) [3]. The most extensive series to date, comprising 41 cases by Hanna et al., reinforced the recognition of CMTCT as a distinct disease entity [4]. Histopathologically, CMTCT is characterized by an assembly of non-pigmented spindle and epithelioid cells, arranged in nests, fascicles, bundles, or sheets. These cells often exhibit a prominent nucleolus and visible mitotic activity, leading to potential diagnostic confusion with clear cell sarcoma (CCS) and metastatic melanoma. The precise biological behavior of CMTCT remains somewhat enigmatic, given the relatively few reported cases and limited duration of follow-up. Here, we report a case of CMTCT with nevus cell clusters morphological characteristics in the dermis of the skin, aiming to contribute to the growing body of knowledge surrounding the histological morphology of this tumor, thereby enhancing its understanding among clinicians and pathologists.

## Case presentation

### Clinical summary

**Fig. 1:**
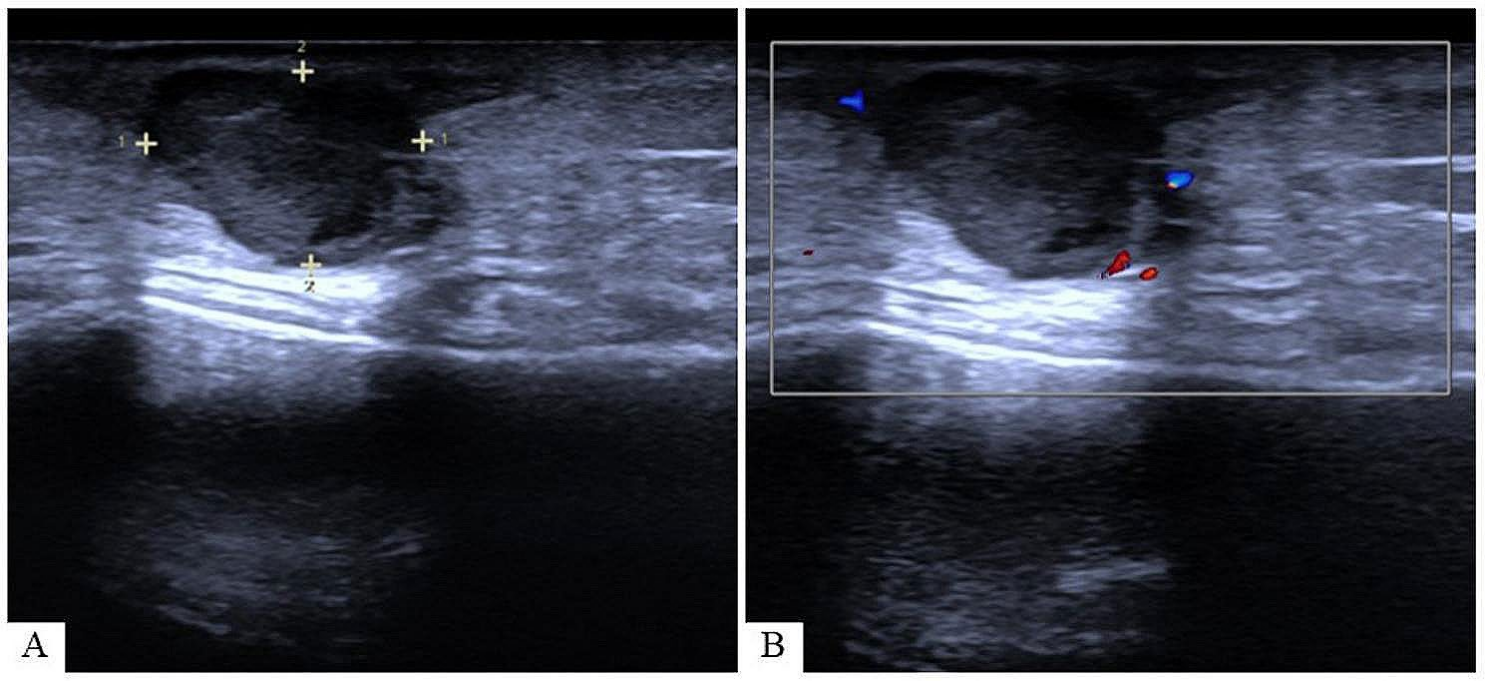
(**A**) A hypoechoic nodule in the subcutaneous soft tissue of the proximal left leg with a distinct boundary, uneven internal echo, and enhanced posterior echo. (**B**) CDFI shows no clear blood flow signal

An 18-year-old female patient noticed a dull red mass on her left leg three years ago, which developed without any clear cause. The mass was occasionally tender and exhibited slow growth. The patient has no family history of tumors, no surgical history, and no chronic diseases. In July 2022, she underwent a B-ultrasound examination at our hospital, revealing a hypoechoic nodule in the subcutaneous soft tissue of the proximal left leg. The nodule measured approximately 1.2 × 1 cm, with a distinct boundary, uneven internal echo, and enhanced posterior echo, CDFI shows no clear blood flow signal. Initially, a benign lesion was suspected, and no significant lymphadenopathy was detected in the bilateral popliteal fossae or groin (Fig. 1). The patient underwent subcutaneous tumor resection, followed by an extended resection due to incomplete initial removal.Postoperative pathology indicated a small residual tumor in the dermis, approximately 0.3 cm in diameter, with negative resection margins. A six-month postoperative follow-up revealed no local recurrence or metastasis.

### Pathological findings of resected specimens

**Fig. 2:**
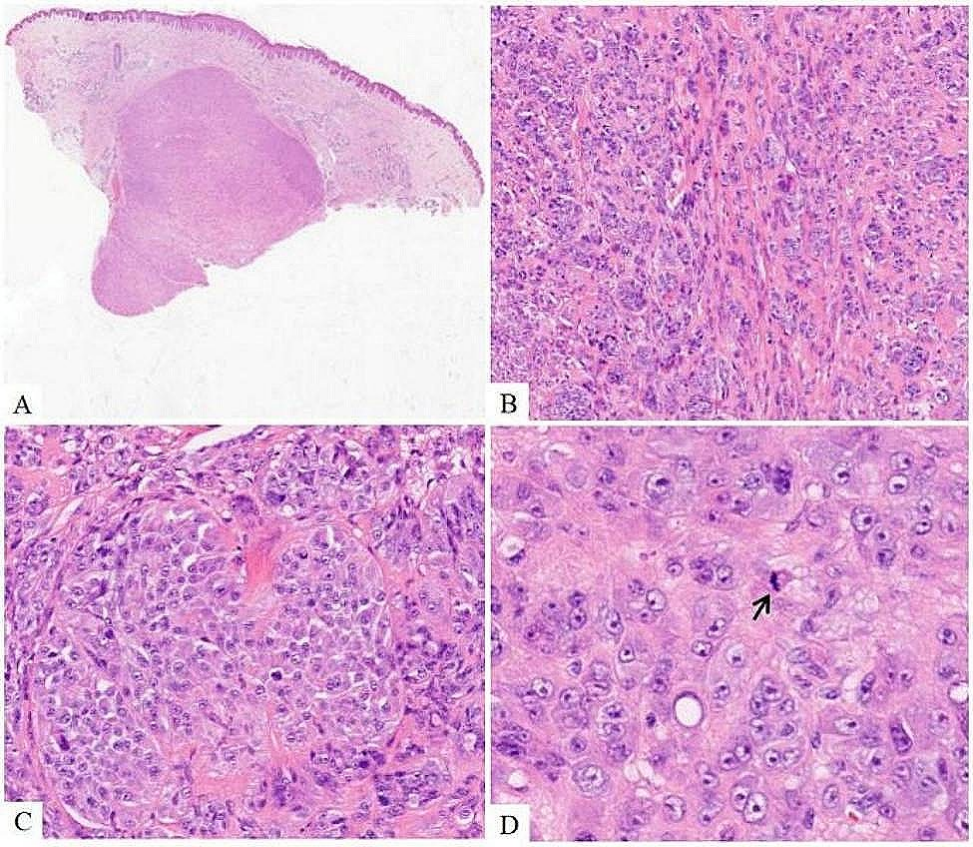
(**A**) Low power magnification shows a circumscribed, unencapsulated dermal tumor(hematoxylin/eosin). (**B**) The neoplasm was composed of nests or sheets of epithelioid cells(×100 hematoxylin/eosin). (**C**) The morphological characteristics of nevus cell clusters can be seen(×200 hematoxylin/eosin). (**D**) The tumor cells showed no pleomorphism, round or oval nuclei, abundant cytoplasm, obvious nucleolus and visible mitosis((×400 hematoxylin/eosin)

**Fig. 3:**
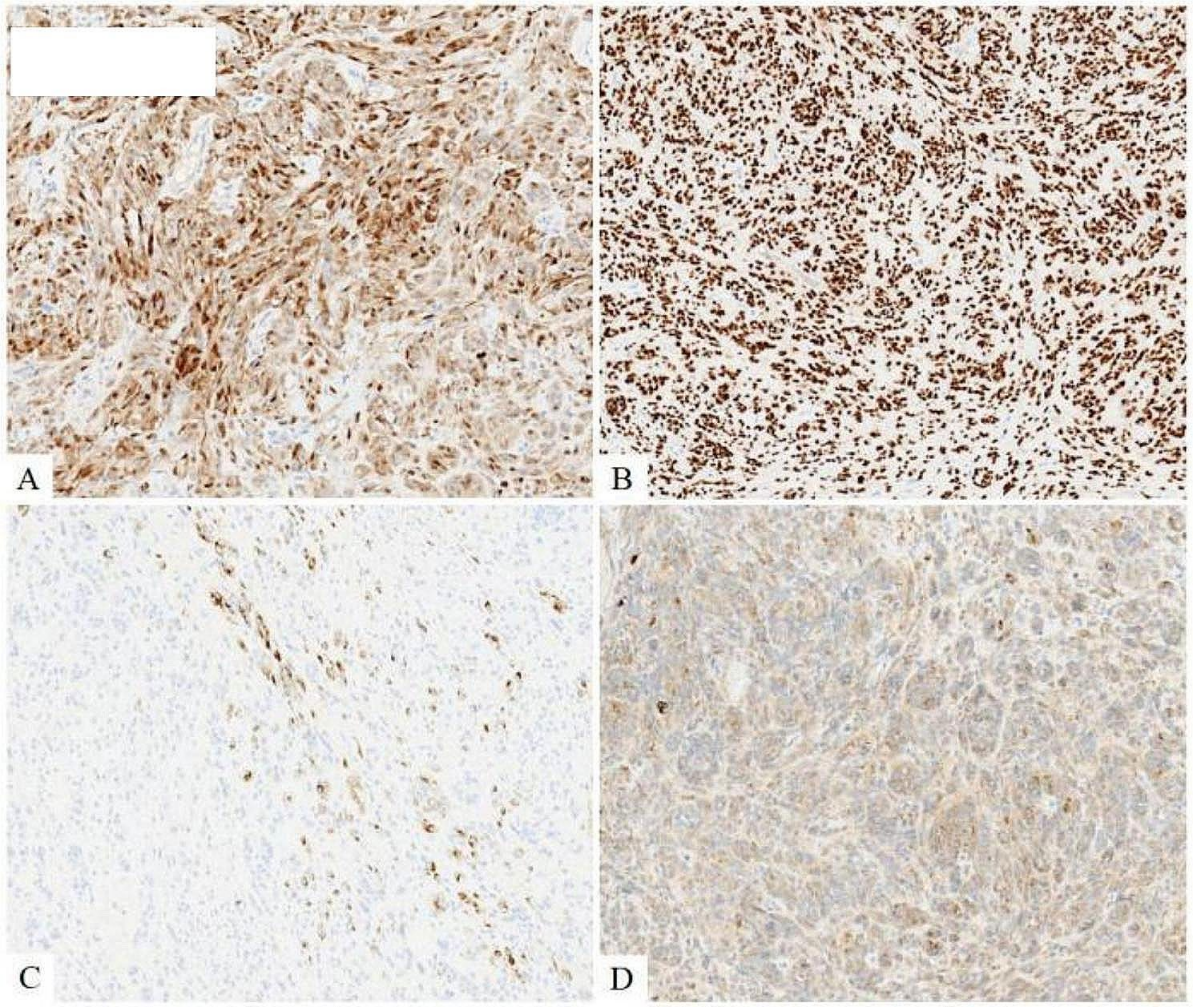
(**A**) S-100 was diffusely positive in cytoplasm(×100 immunohistochemical staining). (**B**) SOX10 was diffusely positive in nucleus(×100 immunohistochemical staining). (**C**) HMB45 is a marker of melanocytic differentiation, HMB45 was locally positive in cytoplasm(×100 immunohistochemical staining). (**D**) Melan A is a marker of melanocytic differentiation. Melan A was diffusely weakly positive in cytoplasm( ×100 immunohistochemical staining)

**Fig. 4:**
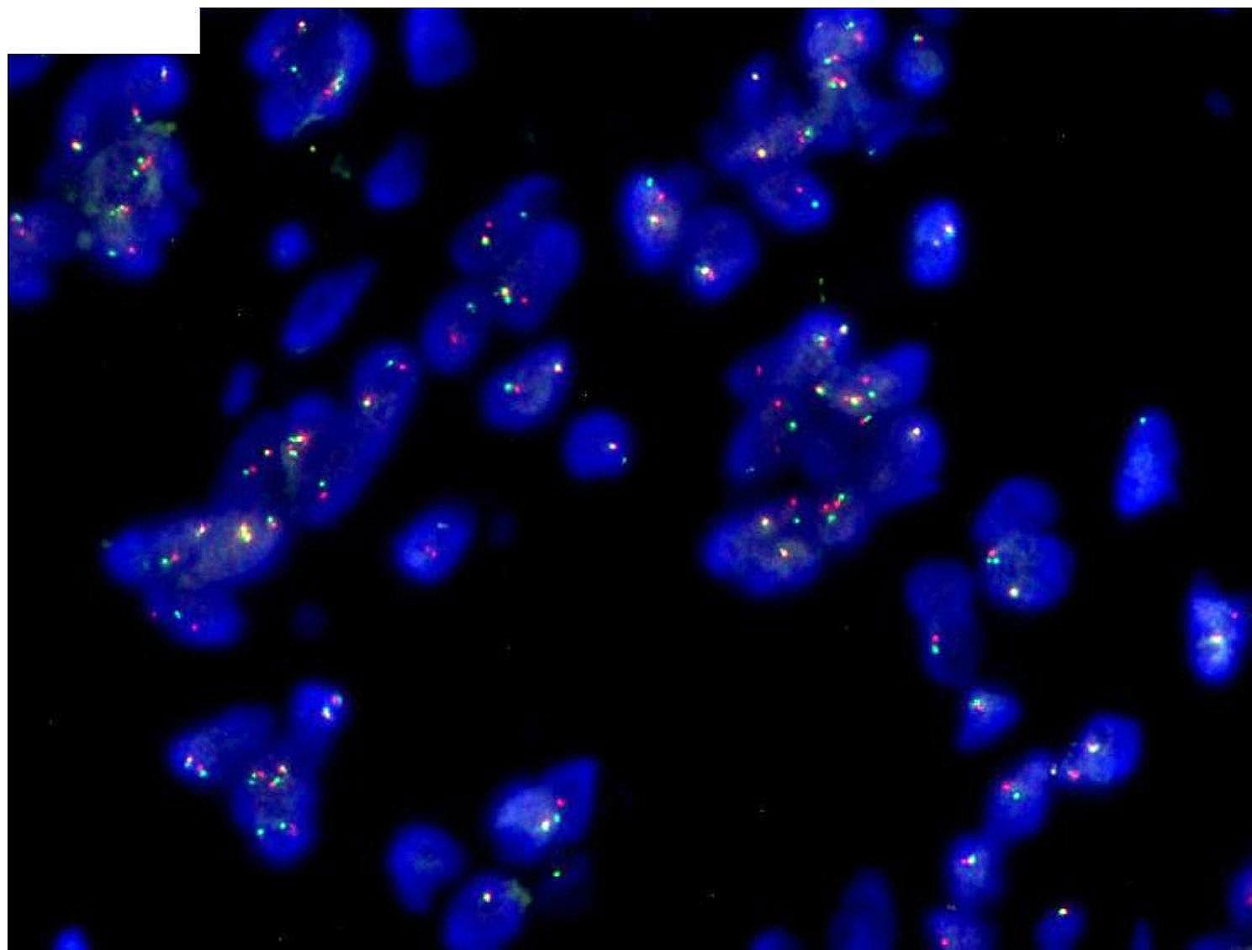
FISH detection result showed that the gene break rearrangement was positive,CRTC1 (red) and the centromere of chromosome 19 (green) in tumor cells showed a two-color separation fluorescence signal

Under low power magnification, a well-circumscribed nodular mass was observed in the dermis. High power magnification revealed the neoplasm to be composed of nests, fascicles, bundles, or sheets of epithelioid cells. The tumor cells displayed round or oval nuclei, abundant cytoplasm, prominent nucleoli, and visible mitosis, with local nevus cell clusters (Fig. 2). The basal margin of the lesion was positive. Immunohistochemistry (IHC) results were positive for S-100, SOX10, MelanA, and focally positive for HMB45. INI1 and H3K27Me3 showed retention of nuclear expression, and the proliferation index hotspot area was approximately 10% (Fig. 3). Initially, the tumor was considered a melanocyte-differentiated neoplasm with local nevus cell clusters. Primary and metastatic melanoma, Clear cell sarcoma (CCS), or malignant transformation of a Spitz nevus were considered as differential diagnoses. However, tests for EWSR1 rearrangement (FISH) and BRAF V600E mutation (PCR-ARMS) were negative. Consequently, we concluded that this was a rare melanocyte-differentiated tumor, distinct from melanoma, CCS, or malignant transformation of Spitz nevus. Next-generation sequencing (NGS) detected a *CRTC1::TRIM11* fusion, and CRTC1 FISH confirmed positive fracture rearrangement (Fig. 4), leading to the final diagnosis of a cutaneous melanocytic tumor with *CRTC1::TRIM11* fusion.

## Discussion and conclusions

Cutaneous Melanocytic Tumor with *CRTC1::TRIM11* Fusion (CMTCT) is a newly identified, rare entity exhibiting melanocytic differentiation. Since its initial description in 2018, the English literature has documented 47 cases, including a recent comprehensive series of 41 cases [1–8]. With the inclusion of our case, the total reported instances now stand at 48. CMTCT typically presents as a slow-growing superficial nodule in various anatomic locations, with the extremities (31/48, 64.6%) being the most common site, followed by the trunk (9/48, 18.7%). Occurrences in other areas like the head and mucosa have also been reported. The patient demographic is predominantly female (28/48, 58.3%), ranging in age from 5 to 87 years, with a median age of 43 years. The tumor size varies from 5 to 51.3 mm, averaging around 11.4 mm, based on data from 17 cases.

Previous literature reports have shown that CMTCT is mostly presented as a well-defined dermal or subcutaneous nodular or multilobulated tumor with pushing borders, only 1 case has reported with prominent epidermal involvement [7]. The neoplasm is composed of nests, fascicles, bundles, or sheets of mostly non-pigmented spindle and epithelioid cells, surrounded by thin collagenous septa. The nucleoli of the tumor cells were obvious, and nuclear division could be seen. Multinucleated giant cells and necrosis may also be identified. Immunohistochemistry showed that the tumor had the characteristics of melanocytic differentiation. Although the expression of S-100, HMB45 and MelanA were different, SOX10 was diffusely positive in all cases. TRIM11 was expressed in nearly all cases tested (94%), TRIM11 IHC may provide an even more accessible approach [4].In this particular case, we identified a distinct nodular mass on the dermis of the left leg with a well-defined boundary. The neoplasm was composed of nests, fascicles, or sheets of epithelioid cells .The tumor cells showed no pleomorphism, obvious nucleolus and visible mitosis with no clear necrosis and multinucleated giant cells, accompanied by nevus cell clusters morphological characteristics locally. Immunohistochemistry (IHC) showed that S-100, SOX10, MelanA were positive, HMB45 was focally positive. Different from previous reports in the literature, this case primarily involved epithelioid cells, with local instances displaying nevus cell clusters characteristics. The tumor also exhibited expression of melanocyte markers, which poses a potential diagnostic challenge, as it can be misinterpreted as cutaneous melanoma. Notably, mature molecular targets for melanoma include BRAF, C-KIT, and NRAS. BRAF mutations, with an incidence of 40-60% in cutaneous melanoma, are the most frequently observed mutations [9, 10]. In our case, the tumor was found to not be connected to the epidermis. The patient discovered a mass on her left leg three years ago, which has a long clinical history and slowly grown of the tumor. The proliferation index of the tumor was low, the cell atypia was minimal, and the BRAF mutation was negative, thus excluding primary or metastatic cutaneous melanoma. Therefore, we suspected Spitz nevus malignant (although a typical Spitz nevus area was not observed), or CCS. Differentiating our case from CCS in terms of histomorphology, clinical manifestations, and location of the disease proved to be challenging. CCS is considered to be morphologically similar to CMTCT and it is difficult to distinguish between the two, it has been reported that 21 of the 41 cases of CMTCT were identified only after molecular analysis for CCS (typically EWSR1 FISH) proved negative [4]. However, CCS often occurs in young adults in the distal tendons and aponeurosis, particularly in the feet, and may be located deeper than CMTCT. Approximately 60% of CCS cases exhibit varying degrees of pigmentation, whereas pigmentation in CMTCT is not prominent. Furthermore, more than 90% of CCS cases are associated with rearrangements of the EWSR1 gene and ATF1, with a few cases involving rearrangements with CREB1 or CREM [11, 12]. In our case, we discovered that the tumor was not CCS, but an unknown type, after negative EWSR1 FISH rearrangement, as well as ruling out primary/metastatic melanoma and Spitz nevus. NGS revealed a *CRTC1::TRIM11* gene fusion, and CRTC1 FISH showed a positive break rearrangement, which just verified our idea. Additionally, the tumor cells in our case exhibited morphological characteristics resembling nevus cell clusters, which were not prominently noted in previous reports. This finding enhances our understanding of the disease’s morphology. Morphologically, CMTCT can be easily mistaken for CCS, primary, or metastatic melanoma. Despite its potential for metastasis, CMTCT appears to be less aggressive than primary or metastatic melanoma and CCS [4]. Therefore, accurate identification of CMTCT is crucial. Molecular detection of *CRTC1::TRIM11* fusion aids in achieving an accurate diagnosis.

CMTCT was considered as a relatively indolent tumor, but it carries some metastatic potential, incomplete resection and deeper tumor location may be associated with poor prognosis. Among the 48 reported cases, 38 showed no recurrence or metastasis during follow-ups ranging from 3 to 168 months. 4 reported cases had recurrence and regional lymph nodes metastases. Of these four cases, two patients had recurrence or metastasis 13 years and 13 months after the lesion was removed [2, 4] .One patient recurred due to incomplete resection of the tumor, after complete resection of the recurrent lesion, the patient was followed up for 20 months without disease progression [4]. One case was a chinese patient, misdiagnosed as intradermal nevus according to morphology after lesion resection. After 18 months, inguinal lymph node metastasis occurred and lymph node resection was performed. Later, the patient suffered recurrent lymph node metastases twice at the same site of right groin and suspected distant lung metastasis, this patient also carried a mutation in the TERT promoter, which the author believes may be related to the more aggressive behavior of CMTCT [8]. In our case, the tumor was not completely resected initially, but subsequent complete resection resulted in no disease progression during follow-up.

In summary, our case of CMTCT with nevus cell clusters morphological features enhances our understanding of this disease’s morphology. Given the limited number of reports, further cases and investigations are necessary to fully elucidate the nature of CMTCT. This case underscores the importance of questioning, consulting literature, and utilizing molecular diagnostics when clinical, microscopic, immunohistochemical, and molecular findings are incongruent in tumors with melanocyte differentiation.

## Data Availability

All data generated or analyzed during this case are included within the article.

## Abbreviations

CMTCT: Cutaneous melanocytic tumor with *CRTC1*:*TRIM11* fusion
WHO: World Health Organization
NGS: Next-generation sequencing
FISH: Fluorescence in situ hybridization
CCS: Clear cell sarcoma
IHC: Immunohistochemistry
pCR-ARMS: *pC*R amplification refractory mutation system
